# The Binding of Brazilin from *C. sappan* to the Full-Length SARS-CoV-2 Spike Proteins

**DOI:** 10.3390/ijms26094100

**Published:** 2025-04-25

**Authors:** Phonphiphat Bamrung, Borvornwat Toviwek, Firdaus Samsudin, Phoom Chairatana, Peter John Bond, Prapasiri Pongprayoon

**Affiliations:** 1 Department of Chemistry, Faculty of Science, Kasetsart University, Chatuchak, Bangkok 10900, Thailand; phonphiphat.b@ku.th (P.B.); toviwek@gmail.com (B.T.); 2 Bioinformatics Institute (BII), Agency for Science, Technology and Research (A*STAR), 30 Biopolis Street, #07-01 Matrix, Singapore 138671, Singapore; mohdfbs@bii.a-star.edu.sg; 3 Department of Microbiology, Faculty of Medicine Siriraj Hospital, Mahidol University, Bangkok 10700, Thailand; phoom.chairat@gmail.com; 4 Department of Biological Sciences, National University of Singapore, Singapore 117543, Singapore; 5 Center for Advanced Studies in Nanotechnology for Chemical, Food and Agricultural Industries, KU Institute for Advanced Studies, Kasetsart University, Bangkok 10900, Thailand

**Keywords:** brazilin, spike protein, MD simulations

## Abstract

The emergence of coronavirus disease (COVID-19) caused by severe acute respiratory syndrome coronavirus-2 (SARS-CoV-2) has become a global issue since 2019. The prominent characteristic of SARS-CoV-2 is the presence of the spike (S) protein protruding from the virus particle envelope. The S protein is a major drug and vaccine target because it initiates the key step in infection. Medicinal herbs are a potential treatment option to enhance immunity to fight viral infections. *Caesalpinia sappan* L. has been reported to display promising anti-viral activities. Specifically, brazilin (BRA), a major bioactive compound in *C. sappan*, was reported to play a role in inhibiting viral infection. Thus, the ability of BRA as a COVID-19 treatment was tested. The S protein was used as the BRA target of this work. Understanding the binding mechanism of BRA to the S protein is crucial for future utilisation of *C. sappan* as a COVID-19 treatment or other coronavirus-caused pandemics. Here, we performed molecular docking of BRA onto the S protein receptor binding domain (RBD) and multimerisation (MM) pockets. Molecular dynamics (MD) simulations were conducted to study the stability of binding to glycosylated and non-glycosylated S protein constructs. BRA can bind to the Receptor-binding motif (RBM) on an RBD surface stably; however, it is too large to fit into the MM pocket, resulting in dissociation. Nonetheless, BRA is bound by residues near the S1/S2 interface. We found that glycosylation has no effect on BRA binding, as the proposed binding site is far from any glycans. Our results thus indicate that *C. sappan* may act as a promising preventive and therapeutic alternative for COVID-19 treatment.

## 1. Introduction

In 2019, the COVID-19 pandemic emerged from Wuhan, Hubei, China [1], caused by a novel coronavirus named severe acute respiratory syndrome coronavirus-2 (SARS-CoV-2) [2,3]. This coronavirus caused a serious outbreak in many countries and territories around the world. SARS-CoV-2 is a large, enveloped, and single-stranded RNA coronavirus belonging to the betacoronaviridae family [4]. The SARS-CoV-2 infection is mediated by the interactions of a transmembrane spike (S) glycoprotein with the human angiotensin-converting enzyme 2 (ACE2) receptor [5,6]. Hence, the S protein is a major drug and vaccine target [7]. When the S protein passes through the cellular secretory pathway, it is glycosylated by the host cellular glycosylation apparatus [8]. This glycosylation plays a vital role in virus–host interactions and immune evasion [8,9] as the glycans can shield underlying epitopes from antibody recognition [10].

**Figure 1:**
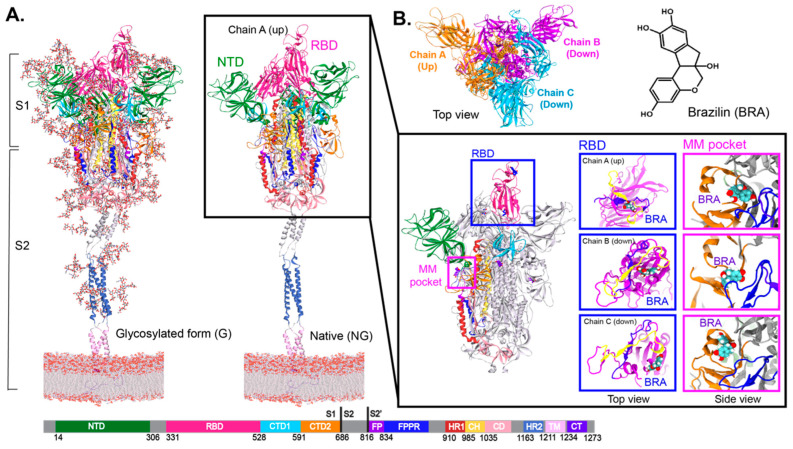
(**A**) Two forms of full-length spike proteins embedded in a membrane. The fully glycosylated form is shown on the left, while the non-glycosylated form is displayed on the right. The inset displays the binding positions of docked brazilin (BRA) inside RBM (blue) and MM pockets (magenta) at 150 ns. The blue violet (residue 443 to 459) and yellow (residue 484 to 505) ribbons show the main contact area between RBM [6] and the ACE2 receptor reported from previous studies [20,21]. (**B**) Top view of homotrimeric spike protein and chemical structure of BRA.

The S protein is a viral fusion protein homotrimer containing two subunits, S1 and S2 (Figure 1A). The S1 subunit contains the receptor-binding domain (RBD), N-terminal domain (NTD), and C-terminal domains 1 and 2 (CTD1 and CTD2), whereas the S2 subunit includes the fusion peptide (FP), fusion-peptide proximal region (FPPR), heptad repeat 1 and 2 (HR1 and HR2), central helix (CH), connector domain (CD), transmembrane segment (TM) and cytoplasmic tail (CT) (Figure 1 and Figure S1B in Supplementary Information). In the S1 subunit, the RBD facilitates host cell recognition by interacting with the ACE2 receptor, while the NTD is a major target for neutralising antibodies [10] (Figure S1 in Supplementary Information). On the other hand, S2 is responsible for viral and host membrane fusion [7]. The RBD-ACE2 receptor interaction has been elucidated by X-ray crystallography [11]. ACE2 was found to align with the receptor binding motif (RBM) on the RBD surface (Figure 1A and Figure S1C in Supplementary Information). The two major conformations of the S protein involve the RBD in an “up” form (receptor-accessible state (open)) and “down” form (receptor-inaccessible state (closed)) (Figure 1). To date, various structures of the S protein in different combinations of RBD-up and -down conformations have been solved by cryogenic electron microscopy (cryo-EM) [6]. These structures are useful for exploring the structural and dynamic properties of both open and closed states. After the engagement of RBD in S1 and the ACE2 receptor on the host cell surface, S1 dissociates upon proteolytic cleavage at a furin cleavage site (RRAR residue 682–685 in Figure 3B). S2 is then refolded into a post-fusion structure to expose the FP, leading to membrane fusion with the host cell [5,12,13].

S protein is the most dominant drug target, whereby many possible druggable pockets have been discovered [6,14,15,16]. Several cryo-EM structures reported cryptic pockets in the RBD and the NTD, which can serve as potential drug targets [15,17,18]. Linoleic acid (LA) and polysorbate (PS) were found in RBD and NTD cryptic sites, respectively [15,17]. PS pocket can also accommodate heme metabolites, biliverdin, and bilirubin [19]. Another approach using MD simulations, whereby the S protein was flooded with benzene molecules to trigger the opening of cryptic pockets, led to the discovery of a novel multimerisation (MM) pocket underneath the 617–628 loop (Figure 1 and Figure S1C in Supplementary Information). This loop is a part of the 630 loop (residue 620–640) that was found to be crucial in maintaining the stability of the S1/S2 interface upon proteolysis [13].

To date, while a number of FDA-approved drugs have been used for COVID-19 treatment [22,23], some herbal remedies have also been suggested as treatment options to enhance immunity to fight viral infections [24]. This preventive treatment and self-defence could act as a primary tool to stop COVID-19 prevalence and severity. Thus, a number of studies are searching for traditional herbs against COVID-19 [2,4,24,25,26,27]. Medicinal herbs serve as useful alternative therapies due to cultural acceptability and minimal side effects [28]. *Caesalpinia sappan* L. (*Leguminosae*) (*C. sappan*) is an important medicinal herb, which has significant socioeconomic and health benefits in developing countries with limited healthcare coverage [29]. *C. sappan* has been reported to display promising anti-viral activities [30,31,32]. Specifically, brazilin (BRA), a major bioactive compound in *C. sappan,* was reported to play a role in inhibiting viral infection [31,32]. Therefore, the ability of BRA as a COVID-19 treatment is investigated. The binding affinities of BRA to S protein were studied here. To explore the microscopic view, molecular docking and molecular dynamics (MD) simulations were performed to understand how BRA interact with the S protein. The binding of BRA to the S protein in “up-down-down” RBD conformation was studied in order to understand the binding affinities of BRA in all possible RBD conformations. BRA was initially docked into the areas of RBM and MM pocket. These sites were chosen because RBM plays a role in ACE2 receptor binding, while the MM pocket shows a comparable site to BRA, and it is adjacent to the 617–628 loop, which is important for S1/S2 stability. The effect of glycosylation of the S protein on BRA binding was also studied. The insights obtained here could benefit future development of therapeutics against COVID-19.

## 2. Results and Discussion

In this work, BRA was docked into the RBD and MM pockets. For RBD-bound BRA, a docked BRA was aligned on the RBD-ACE2 surfaces (Receptor-binding motif (RBM)) close to the main interface reported by previous studies [20,21] (insets in Figure 1A). In the case of MM-bound BRA, it was docked inside the hydrophobic binding pocket discovered via a combination of simulations and hydrogen-deuterium mass spectrometry in a previous study [6]. However, the docked BRA is too big to fit in such a hydrophobic pocket, thus, it escapes to a region near the S1/S2 cleavage site, CTD2, FP, and FPPR domains (Figure 1A and Figure S1B in Supplementary Information). More in-depth details will be explained later in the text. The “MM site” will be used to refer to the BRA-binding area close to the MM pocket. Comparing among chains, the docked BRA in chain C seems to reside in different areas of the pocket (Figure 1A). This is because the S1 conformation of chain C slightly differs from the others. Chain C sits next to RBD-up chain A, whose RBD is packed close to the RBD-up of chain A. This pose causes the outward movement of FPPR and furin site, as seen in a previous work [33] leading to different MM and RBD pocket environments. On the contrary, the binding pockets of chains A and B are similar. The presence of glycans does not interfere with the binding of docked BRA to the RBD and MM pockets. For the same corresponding pocket, BRA in both glycan (G) and non-glycan (NG) systems are aligned in the same locations.

**Figure 2:**
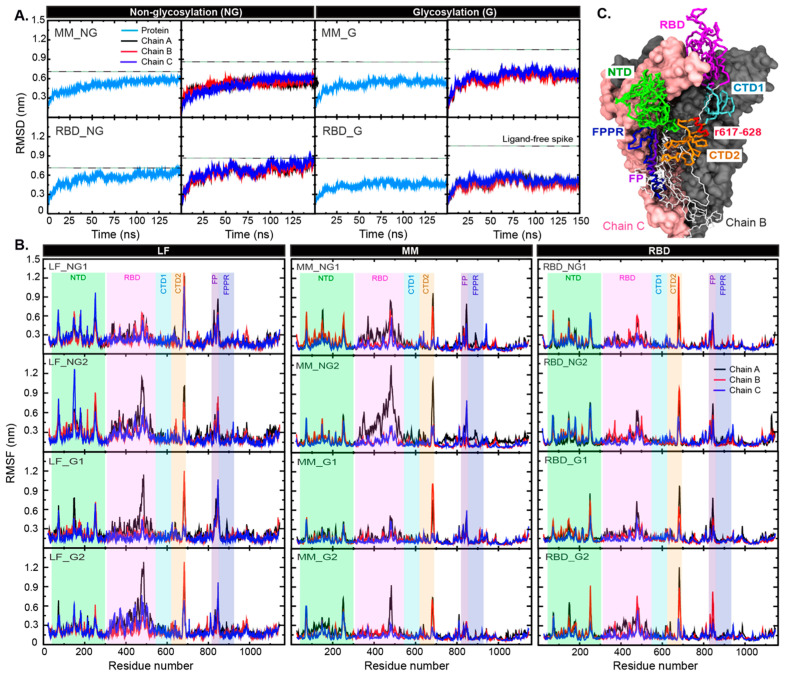
(**A**) C-alpha RMSDs of the whole protein and each chain in all systems. The dashed lines stand for RMSD values of ligand-free (LF) S protein obtained from a previous work [6]. (**B**) RMSFs of each chain in all systems. The RMSF data of LF from a previous work [6] are displayed on the left for comparison. The green, pink, cyan, orange, purple, and blue bands refer to the regions that show high structural fluctuation. The locations of such regions are displayed in (**C**). Chain A is shown in the trace format, whereas chains B and C are in van der Waals surfaces. Key domains are also labelled. Residues 617–628 around the MM pocket are shown in red trace.

To explore how BRA affects the structural flexibilities of the S protein, the simulated root mean-square deviation (RMSD) and fluctuations (RMSFs) of all C-alpha atoms were computed in Figure 2. The initial structure at time = 0 ns was used as a reference for RMSD. The RMSDs of the ligand-free S protein systems are also displayed in Figure 2. These data were obtained from a previous work [6]. Compared to ligand-free wild-type S protein (LF), the presence of bound-BRA in either RBD or MM sites can enhance the protein rigidity (Figure 2). In particular, the glycosylated forms (MM_G and RBD_G) become less mobile (Figure 2A). Each individual monomer displays similar degrees of structural flexibility. To investigate the source of structural fluctuations, RMSFs were also computed (Figure 2B). In LF, the NTD and RBD act as the main contributors to the protein flexibility. The presence of BRA appears to reduce the flexibility of NTD and RBD significantly in the glycosylated S protein systems (Figure 2B). Similarly, high fluctuations of loop regions (residue 668–698 and 800–850) observed in LF are reduced in BRA-bound systems, especially when bound to the MM pocket (Figure 2B and Figure S1 in Supplementary Information for the loop locations). Within these loop regions lie residues 682–685 (RRAR), which form the furin cleavage site [33,34] (see Figure S1A in Supplementary Information for the location). Increased rigidity of this region upon BRA binding could potentially block the accessibility of proteases. Interestingly, the binding of BRA to the MM site also enhances the RBD mobility of chain A (RBD-up) in the non-glycosylated S protein (Figure 2B), which may potentially interrupt ACE2 receptor interaction.

**Figure 3:**
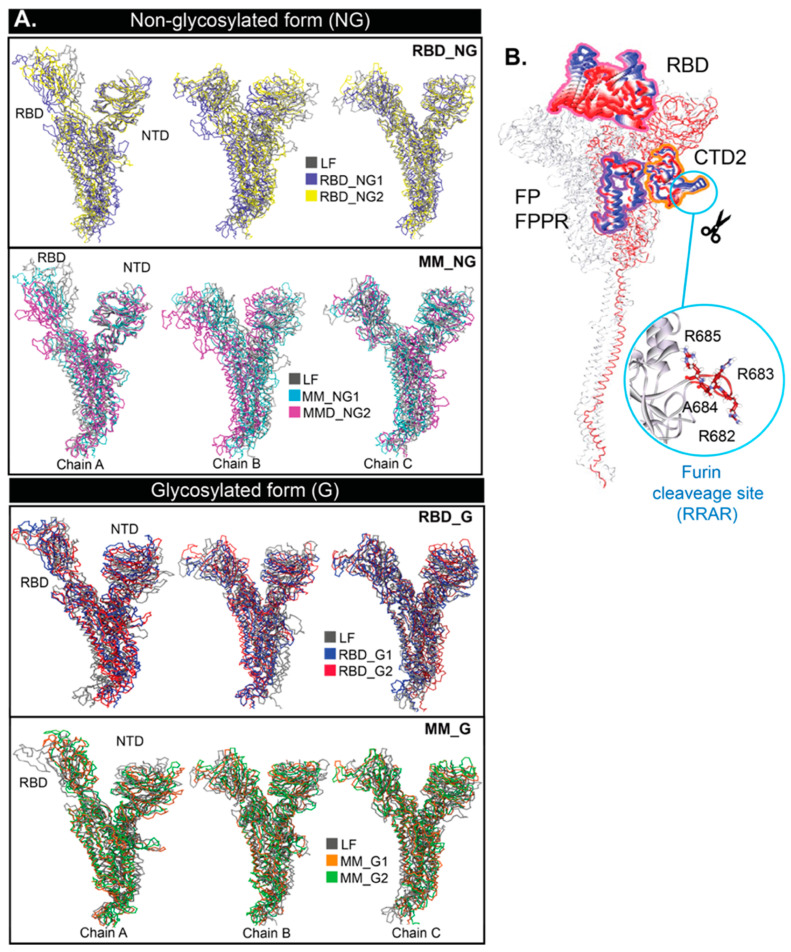
(**A**) Superimpositions of ligand-free (LF) and BRA-bound core structures at 150 ns. (**B**) Principal component analysis (PCA) of the full-length S protein calculated from the first principal component of all systems. Only highly mobile regions are shown in RWB format. Residues forming the furin cleavage site (R682, R683, A684, and R685) are also displayed as an inset.

To explore how the binding of BRA interferes with S protein flexibility, we aligned the final snapshots of LF and BRA-bound S protein and performed principal component analysis (PCA) (Figure 3). Compared to LF, RBD appears to shift outwards upon BRA binding. Especially, the clear outward shift in RBD-up of chain A is captured in both glycosylated (G) and non-glycosylated (NG) states (Figure 3A). The superimposition of all systems can be seen in Figure S3 in the Supplementary Information. PCA confirms the movement of the RBD (Figure 2B). PCA of all systems can be seen in Figure S4 in the Supplementary Information. Apart from RBD, the CTD2, FP, and FPPR also display some mobility upon BRA binding (Figure 2B). The furin cleavage site (residue 682–685) also displays a high mobility (Figure 3B), which is in good agreement with a previous work [35]. Also, the furin site (RRAR) shows no significant interaction with glycans in all cases (Table S2 in Supplementary Information) because of no nearby glycans. However, recent studies have reported an O-glycosylation site nearby to the furin cleavage site in variants of concern (VOCs), which was not considered here, may potentially interfere with the furin activities, resulting in the disruption of viral infection [36,37,38].

To understand the binding mechanisms of BRA in both RBD and MM sites, hydrogen bond analysis was performed. The high number of BRA-water hydrogen bonds (~4–5 hydrogen bonds) demonstrates the wettability of the RBD and MM sites (Table 1). BRA in the non-glycan MM system (MM_NG) shows more water contacts than MM_G. Although BRA exits the hydrophobic MM pocket, it is trapped at the mouth of a pocket (MM site) and forms higher protein interactions than the RBD site (Table 1). The interaction energies show that the BRA binding in both locations is driven by hydrophobic interactions, especially the MM site (Table 2). The total energies confirm that the MM site is more preferable for BRA than the RBD site (Table 2). In case of glycans, RBM is glycan-free, while the MM site is close to one glycan site where no significant glycan-BRA hydrogen bonds are found (Figure S2 and Table S4 in Supplementary Information). This suggests that glycosylation does not affect BRA binding affinities to the wild-type spike protein.

**Figure 4:**
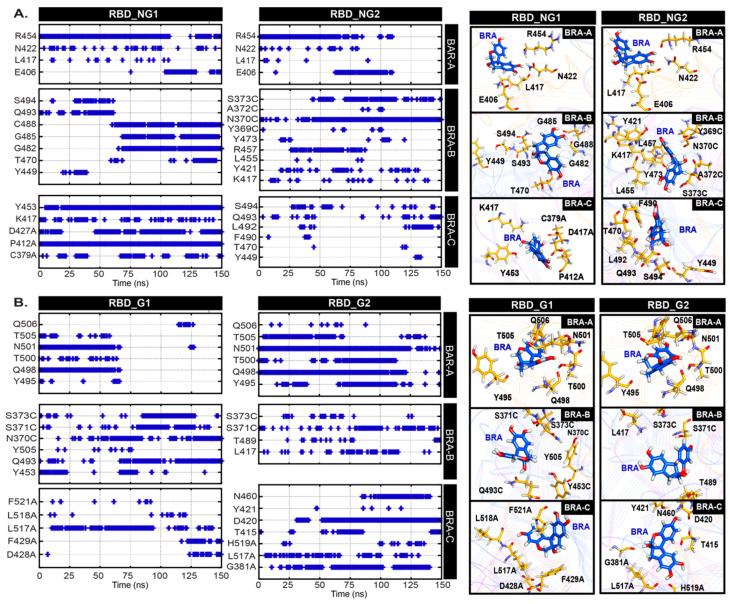
Occurrence frequencies of hydrogen bonds between BRA and cavity-lining residues inside the RBD pocket as a function of time in (**A**) non-glycosylated (NG), and (**B**) glycosylated (G) systems. Only interactions that show ≥50% contacts are investigated here. BRA and key residues inside a pocket are shown on the right.

**Figure 5:**
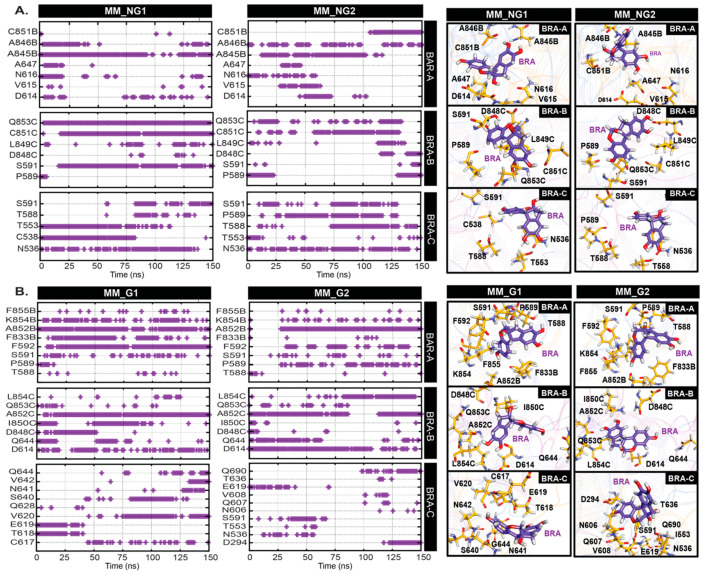
Occurrence frequencies of hydrogen bonds between BRA and cavity-lining residues inside the MM pocket as a function of time in (**A**) non-glycosylated (NG), and (**B**) glycosylated (G) systems. BRA and key residues inside the pocket are shown on the right.

In addition, we mapped the hydrogen bond networks to reveal possible binding mechanisms of BRA (Figure 4 and Figure 5). As the RBD pocket is more water-accessible, this results in displacement of BRA from the RBD surface (Figure S5 in Supplementary Information). Although BRA in both RBD_G and RBD_NG are aligned to the RBD-ACE2 interface or RBM (see Figure S5 for the location), the high-water accessibility allows diverse BRA-residue interaction patterns. BRA in chain A (RBD-up) appears to bind permanently to RBM in both glycan and non-glycan systems via interactions with E406, L417, N422, and R454 in RBD_NG and Y495, Q498, T500, N501, T505, and Q506 in RBD_G (Figure 4). In RBD_NG2 and RBD_G1, the displacement of BRA is captured, resulting in the loss of interactions, as shown in Figure 4. BRA in RBD_NG2 was found to diffuse to the upper part of RBM and stay there until the end of all simulations (Figure 4A), whereas BRA in RBD_NG1 moved out of the RBM area (Figure S5A,B in Supplementary Information). Although some BRAs displace to non-RBM areas, they still interact with non-RBM residues that were previously reported to be important for ACE2 receptor binding affinity [39], suggesting that it might still be able to interrupt interaction with ACE2. In the case of RBD-down chains (chain B and C), BRA appears to be trapped in a cavity between RBM and the side of the RBD core of the adjacent monomer (Figure S5C in Supplementary Information), which allows BRA to interact with residues in the adjacent unit. These binding areas are the interface between the cryptic pocket that binds to linoleic acid [15] (see Figure S6 for locations).

The MM pocket is located in a region containing the 617–628 loop [6] (Figure 2C), whose conformational state affects the structural stability of the S1/S2 interface and hence may influence the premature shedding of the S1 subunit upon proteolysis [40]. Our docking study reveals that the MM pocket is too small to accommodate BRA, thus the most favourable BRA location obtained from docking is an area close to the 617–628 loop (Figure S7B in Supplementary Information). Interestingly, BRA remained bound to the S protein throughout the course of the simulations. Although BRA cannot reside underneath the 617–628 loop due to its large size, BRA is bound by nearby regions between S1 and S2 interface ((residues 536–538 (S1), 586–591 (S1), and 848–850 (S2 from its adjacent monomer)) (see locations in Figure S7B in Supplementary Information) in most cases. In particular, the 848–850 loop from the adjacent chain was found to move upward in order to facilitate stable BRA binding (Figure S7B in Supplementary Information). Only BRA in chain C of MM_G2 was found to translocate away from the 617–628 loop (Figure S7A in Supplementary Information). In general, BRA forms hydrogen bonds with diverse residues in three regions (residue 536–538 (S1), 586–591 (S1), and 848–850 (S2 from its adjacent monomer)). Most BRAs in chain C of both glycan (G) and non-glycan (NG) systems seem to be shifted upwards, resulting in the loss of interactions with the S2 subunit (Figure 5). BRA in MM_NG move upwards and interact with N536, C538, T553, T588, P589, and S591. Unlike MM_NG, BRA in MM_G is not only shifted upwards, but it is also moved close to the 627–628 loop. This movement allows BRA in chain C of MM_G to hydrogen bond with residues nearby in the 617–628 loop (C617, T628, E629, V620, S640, N641, V642, and Q644) (Figure 5). Instead, BRA in chain C of MM_G2 appears to translocate towards the NTD and forms interactions with D294, N606, Q607, V608, and Q690, respectively (Figure 5) (location can be seen in Figure S7A). In the case of BRAs in chain A and B, they are found to interact with N536, C538, D614, V615, N616, and A647 on the regions of the S1 subunit and residues 845–855 on the S2 subunit (Figure S7B in Supplementary Information). Interestingly, BRAs in some cases can interact with D614 (Figure 5A). The D614G mutation has been associated with increased helicity of the 617–628 loop, which contributes to a more stable S1/S2 interface, leading to increased infectivity of the virus [38,41,42]. The BRA binding here seems to slightly enhance the helicity content of the 617–618 loop (Table S5 in Supplementary Information). Nonetheless, the BRA–D614 interactions found here suggest the potential ability of BRA to interfere the infectivity. Although the displacement of BRA was captured in MM systems, the consistent interaction energies suggest that BRA is stabilised by an interaction network with the S protein throughout the course of simulations (Figure S8 in Supplementary Information).

## 3. Materials and Methods

The trimeric structures of full-length glycosylated (G) and non-glycosylated (NG) spike proteins embedded in a membrane (47% phosphatidylcholine (PC), 20% phosphatidylethanol amine (PE), 11% phosphatidylinositol phosphate (PIP), 7% phosphatidylserine (PS) and 15% cholesterol) were obtained from a previous study [6]. The RBD in one monomer (chain A) is in the “up” conformation, whereas the other two (chain B and C) are in the “down” state (Figure 1). For the glycosylated form, there are 22 N-glycosylation sites on each subunit of the S protein (Figure 1A and Figure S2 in Supplementary Information). The most dominant glycan on each site, based on mass spectrometric data was used [43]. The structure of brazilin (BRA) was obtained from PubChem [44] and the topology was generated using the CHARMM general force field (CGenFF) [45]. BRA was then docked into the RBM and MM pockets using GOLD5.3 software [46,47] with default settings. Residues lining the pockets used for docking were defined based on previous studies [6,21]. Residues within a distance of 10 Å from a ligand were defined as the binding site. No water was included in this docking step. The ligand-flexible docking was conducted. The spike-BRA complexes showing the highest GOLD-score in each form (glycosylated (G) and non-glycosylated (NG) forms) were used for further MD studies. In total, there are four BRA-spike systems (MM-NG, MM-G, RBD-NG, and RBD-G). MM and RBD refer to the BRA positions at MM and RBD sites, respectively, whereas NG and G stand for the glycosylation states. Each BRA-spike complex was solvated in a TIP3P water box with dimensions of 23.5 × 23.5 × 40 nm^3^. Each system was neutralised with 0.15 M NaCl.

All simulations were performed using the GROMACS 2020 package (http://www.gromacs.org) [48] using the CHARMM36m [49] force field. Long-range electrostatic interactions were treated using the particle mesh Ewald (PME) algorithm with a real-space cutoff of 1.2 nm and a Fourier spacing of 0.16 nm. A 1000-step energy minimisation was performed to relax steric conflicts using the steepest descent algorithm. Then, a 10 ns equilibration run was performed in all systems, followed by a 150 ns production run. All simulations were performed in the constant number of particles, pressure, and temperature (NPT) ensemble. Protein, ligands, solvent and ions were coupled separately using the Nosé-Hoover thermostat [50,51] at 310 K with a coupling constant τ_t_ = 1 ps. The pressure was coupled using the Parrinello−Rahman barostat at 1 bar with a coupling constant τ_p_ = 5 ps using semi-isotropic pressure coupling. The time step for integration was 2 fs. Each system was repeated twice (the suffixes of “1” and “2” are used to represent simulations 1 and 2). In total, eight simulations were thus performed (MM_NG1, MM_NG2, MM_G1, MM_G2, RBD_NG1, RBD_NG2, RBD_G1, and RBD_G2). The BRA-bound spike simulations were studied in comparison to the ligand-free spike protein (LF) systems from previous work [6].

All results provided here are the average values from two simulations. The data were analysed using GROMACS and locally written code. All graphical images were prepared using VMD [52] and the Discovery studio. The C-alpha RMSD calculations were computed using the initial structure at t = 0 ns as a reference. For principal component analysis (PCA), it was calculated by the default “gmx covar” and “gmx anaeig” options in GROMACS. For hydrogen bond calculation, the hydrogen-donor-acceptor cutoff angle was 30°, and the cutoff radius (X-acceptor) of 0.35 nm. The binding energies of BRA-spike were computed using “gmx mmpbsa”.

## 4. Conclusions

In this work, we studied the potential binding mechanisms of BRA to the S protein in both non-glycosylated and glycosylated forms. We found that BRA can bind to the RBM region, but it does not fit inside the MM pocket due to its large size. The binding of BRA to the RBM suggests the ability of BRA to block RBD-ACE2 binding. In the case of the MM pocket, even though BRA dissociates from the MM pocket in all systems due to the large size, it seems to bind close to the S1/S2 cleavage site. BRA is bound by regions close to the 630 loop. Importantly, BRA can interact with residue D614, whose mutation has been associated with increased viral fitness; hence, it is possible that BRA is able to interfere with viral infectivity. Glycosylation appears to have no effect on BRA binding because it binds to the glycan-free area. Collectively, our results indicate that *C. sappan* could represent a promising lead for preventive or therapeutic alternatives for COVID-19 treatment.

## Figures and Tables

**Table 1:** Average number of BRA–water and BRA–protein hydrogen bonds with standard deviations.

System	Number of Hydrogen Bonds					
	Water			Protein		
	BRA-A	BRA-B	BRA-C	BRA-A	BRA-B	BRA-C
MM_NG1	5.6 ± 1.5	4.6 ± 1.4	5.5 ± 1.6	1.5 ± 1.1	2.48 ± 0.73	1.3 ± 0.9
MM_NG2	5.4 ± 1.5	4.9 ± 1.6	4.9 ± 1.5	1.3 ± 0.9	1.53 ± 1.18	1.9 ± 1.1
MM_G1	4.0 ± 1.3	3.7 ± 1.4	4.0 ± 1.7	1.5 ± 0.8	1.66 ± 0.87	1.3 ± 0.9
MM_G2	4.4 ± 1.4	4.1 ± 1.6	5.6 ± 1.5	1.2 ± 0.8	2.14 ± 1.15	0.8 ± 0.7
RBD_NG1	5.2 ± 1.3	5.1 ± 1.7	3.8 ± 1.3	0.7 ± 0.6	1.46 ± 1.07	2.4 ± 0.8
RBD_NG2	5.0 ± 1.4	4.8 ± 1.6	5.8 ± 1.5	0.8 ± 0.6	1.04 ± 0.76	0.5 ± 0.7
RBD_G1	5.7 ± 1.5	4.4 ± 1.7	5.8 ± 1.5	1.5 ± 1.0	1.18 ± 0.89	0.6 ± 0.6
RBD_G2	4.4 ± 1.6	4.7 ± 1.5	4.4 ± 1.6	2.4 ± 1.4	0.39 ± 0.59	1.8 ± 0.9

**Table 2:** Interaction energies (kJ/mol) between BRA and spike protein were calculated using the MMPBSA method using the last 100 ns of the simulations. Only the average values are shown. The data with standard deviation can be seen in Table S3 in the Supplementary Information. BRA-A, BRA-B, and BRA-C refer to BRA bound to chains A, B, and C, respectively.

System	BRA-A			BRA-B			BRA-C		
	ΔE_vdw_	ΔE_Elec_	Total Energy	ΔE_vdw_	ΔE_Elec_	Total Energy	ΔE_vdw_	ΔE_Elec_	Total Energy
MM_NG1	−127.6	−45.3	−172.9	−125.0	−120.4	−245.4	−63.4	−83.0	−146.3
MM_NG2	−106.3	−45.4	−151.7	−76.5	−96.5	−173.0	88.0	−67.0	−155.0
MM_G1	−126.7	−93.1	−219.8	−102.8	−46.8	−149.6	−77.4	−49.6	−127.0
MM_G2	−102.7	−83.0	−185.7	−122.3	−132.0	−254.3	−83.3	−59.6	−143.0
RBD_NG1	−88.8	−70.3	−159.1	−85.6	−71.3	−156.9	−109.3	−139.5	−248.8
RBD_NG2	−81.6	−44.5	−126.2	−122.0	−38.9	−160.9	−71.4	−15.7	−87.1
RBD_G1	−51.8	−29.3	−81.1	−59.1	−53.5	−112.6	−77.4	−37.0	−114.4
RBD_G2	−83.5	−50.8	−134.4	−90.2	−20.0	−110.2	−106.4	−116.5	−222.9

## Data Availability

All important data are included in the manuscript.

## Supplementary Materials

The following supporting information can be downloaded at: https://www.mdpi.com/article/10.3390/ijms26094100/s1. References [14,15,53] are cited in the Supplementary Materials.

## Abbreviations

The following abbreviations are used in this manuscript:

MD	Molecular Dynamics
BRA	Brazilin
