# Supplementary material for: The Binding of Brazilin from C. sappan to the Full-Length SARS-CoV-2 Spike Proteins
Source: Int J Mol Sci. 2025 Apr 25;26(9):4100. doi: 10.3390/ijms26094100 (PMC12072004; doi:10.3390/ijms26094100)
Supplement: Supplementary file 1 [file ijms-26-04100-s001.zip › ijms-3592711-supplementary.pdf]

Supplementary Information

The binding of brazilin from *C. sappan* to the full-length SAR-CoV-2 spike proteins

Phonphiphat Bamrung[1#], Borvornwat Toviwek[1#], Firdaus Samsudin[2], Phoom Chairatana[3], Peter John Bond[2,4*], and Prapasiri Pongprayoon[1,5*]

[1]Department of Chemistry, Faculty of Science, Kasetsart University, Chatuchak, Bangkok, 10900, Thailand.

[2]Bioinformatics Institute (BII), Agency for Science, Technology and Research (A*STAR), 30 Biopolis Street, #07-01 Matrix, Singapore 138671, Republic of Singapore

[3]Department of Microbiology, Faculty of Medicine Siriraj Hospital, Mahidol University, Bangkok, 10700, Thailand

[4]Department of Biological Sciences, National University of Singapore, Singapore 117543, Singapore

[5]Center for Advanced Studies in Nanotechnology for Chemical, Food and Agricultural Industries, KU Institute for Advanced Studies, Kasetsart University, Bangkok 10900, Thailand.

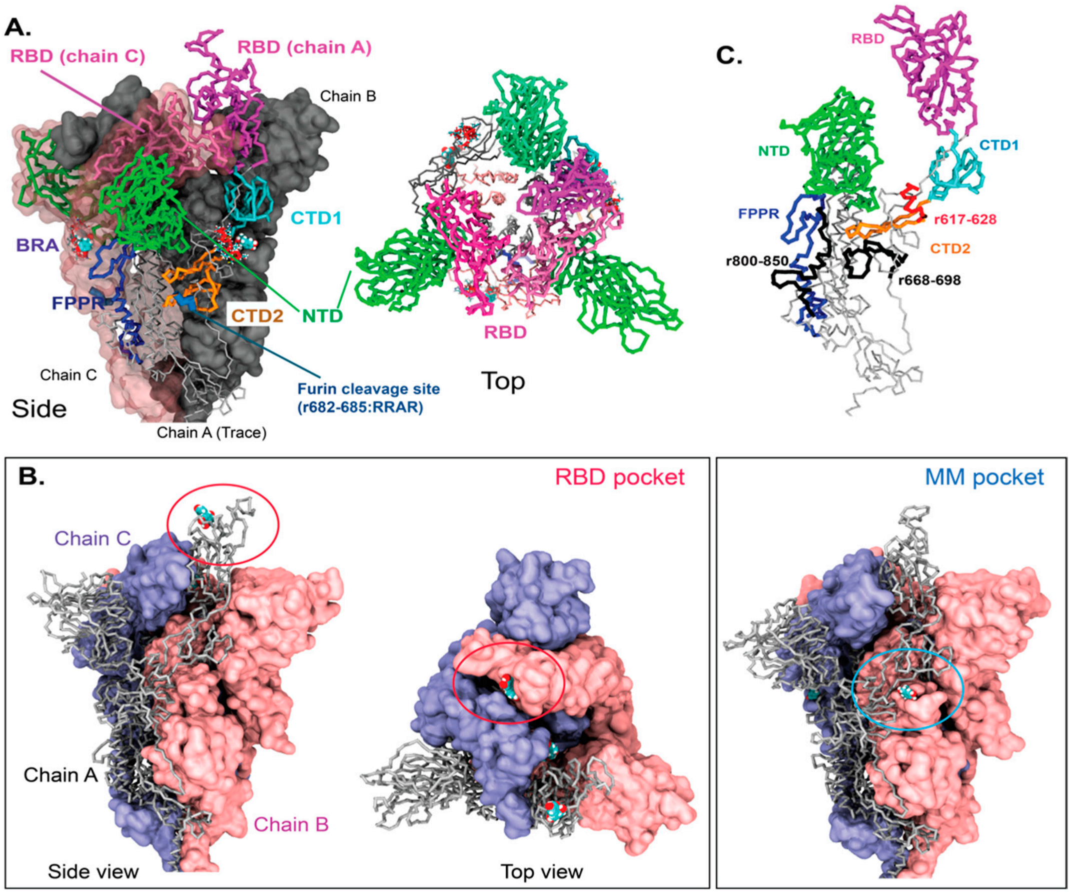

Figure S1. (A) Side and top views of trimeric spike protein (chain A-C). Chain A is in the RBD-up conformations and chain B and C are in RBD-down conformation. Key domains are

coloured and labelled. Chain A is shown in “trace” format, while chain B and C are displayed in gray and pink in van der Waals surfaces. (B) Locations of BRA in the RBD and MM pockets. (C) Monomeric structure of spike protein with colored domains. Residues showing high fluctuation are shown in black (residue 668-698 and 800-850).

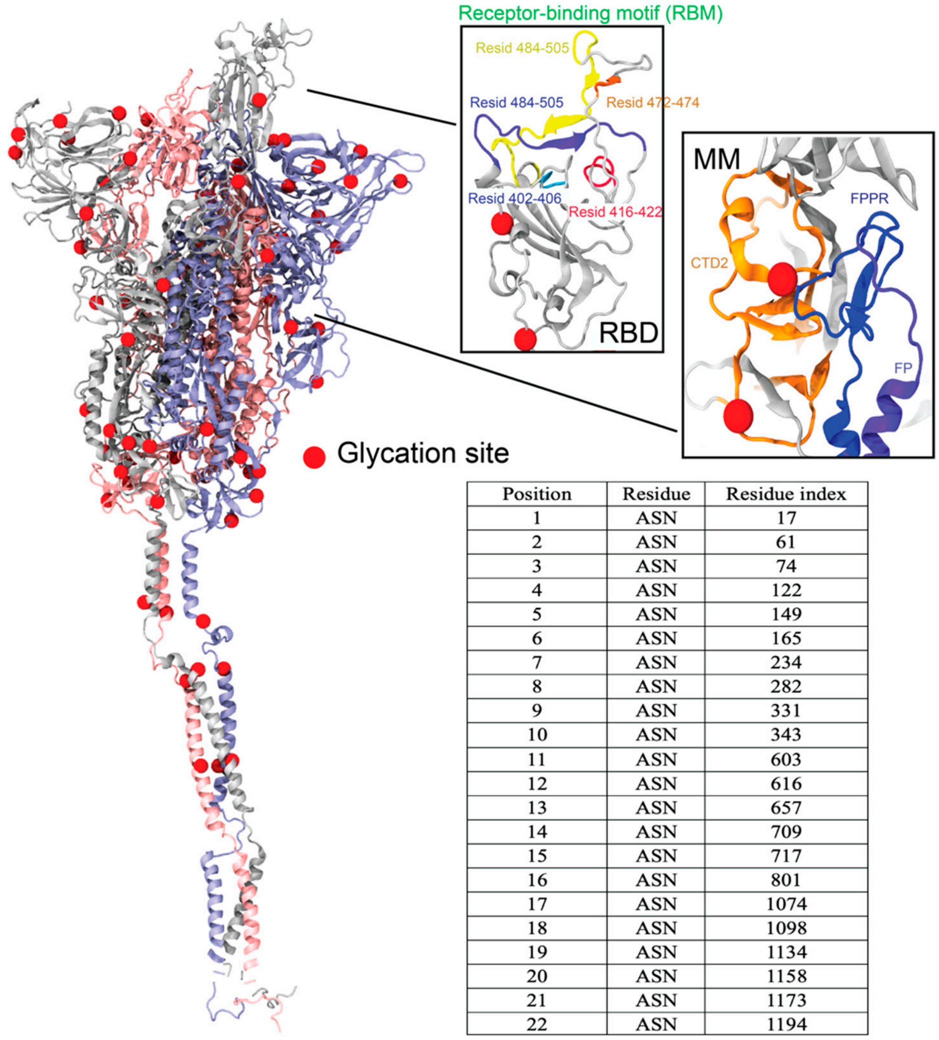

Position	Residue	Residue index
1	ASN	17
2	ASN	61
3	ASN	74
4	ASN	122
5	ASN	149
6	ASN	165
7	ASN	234
8	ASN	282
9	ASN	331
10	ASN	343
11	ASN	603
12	ASN	616
13	ASN	657
14	ASN	709
15	ASN	717
16	ASN	801
17	ASN	1074
18	ASN	1098
19	ASN	1134
20	ASN	1158
21	ASN	1173
22	ASN	1194

Figure S2. (Left) Trimeric structure of spike protein where the glycosylation sites on spike protein is shown by the red dot. (Right) Residues that are reported to be ACE2 binding site are shown in yellow, blue, orange, cyan, and red, whereby yellow and blue ribbons are the major ACE2 contact areas. The residues that are glycosylated are shown in the table.

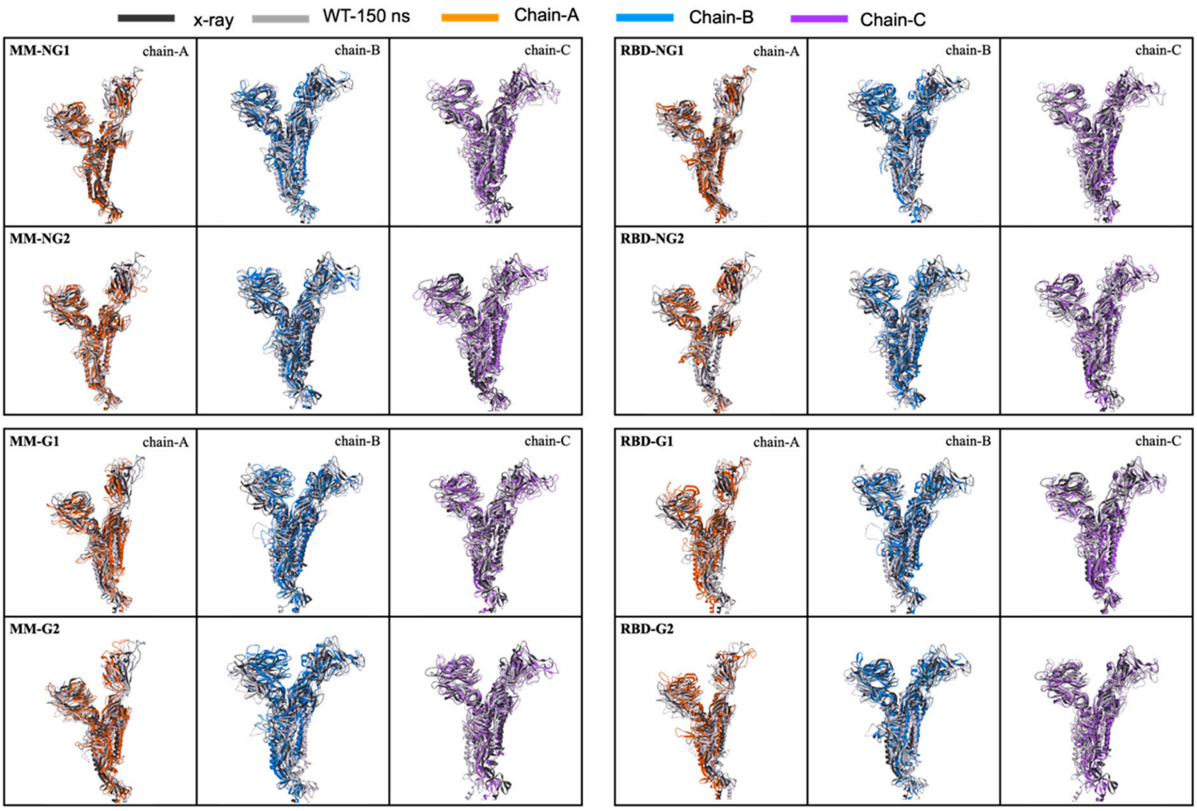

Figure S3. Superimpositions of cryo-EM structure (PDB code: 6VSB), native wild-type (WT) at 150 ns, and spike proteins from each system.

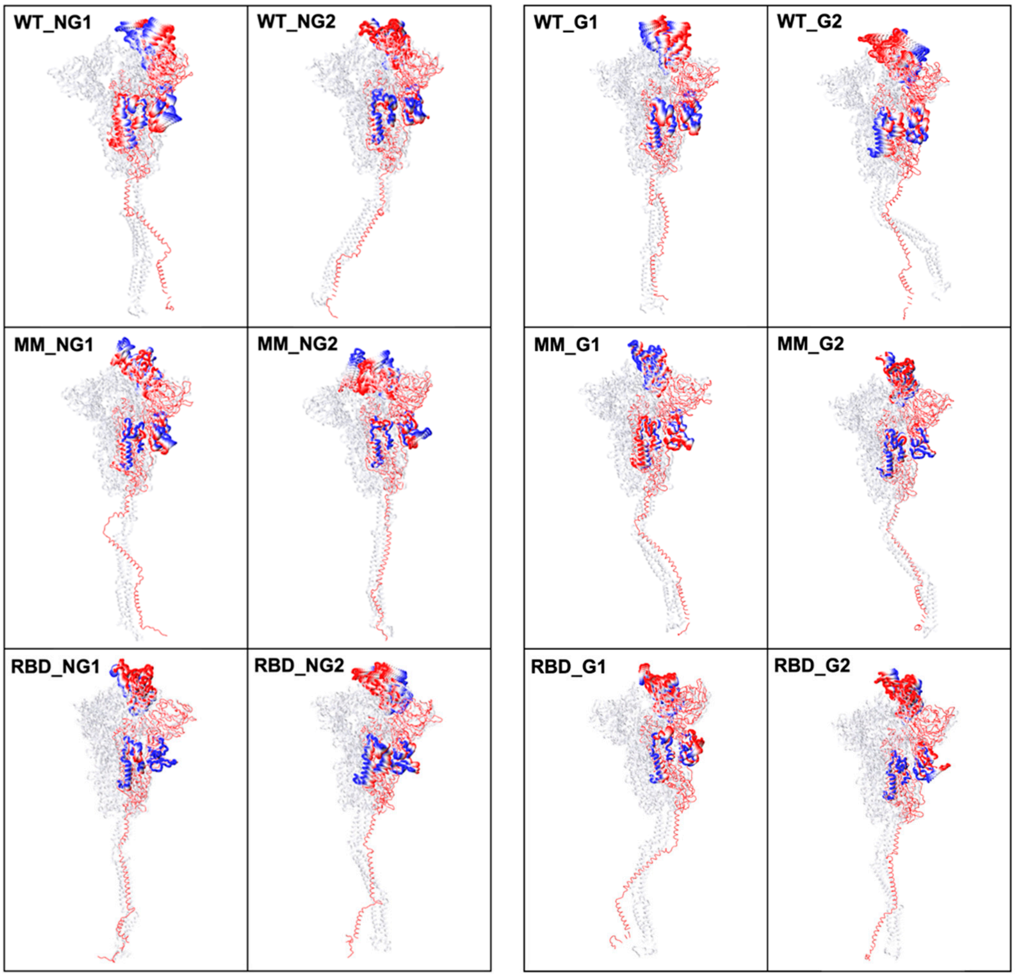

Figure S4. Principal Component Analysis (PCA) of the full-length spike protein calculated from the first principal component of all systems. Only highly mobile regions are shown in RWB format.

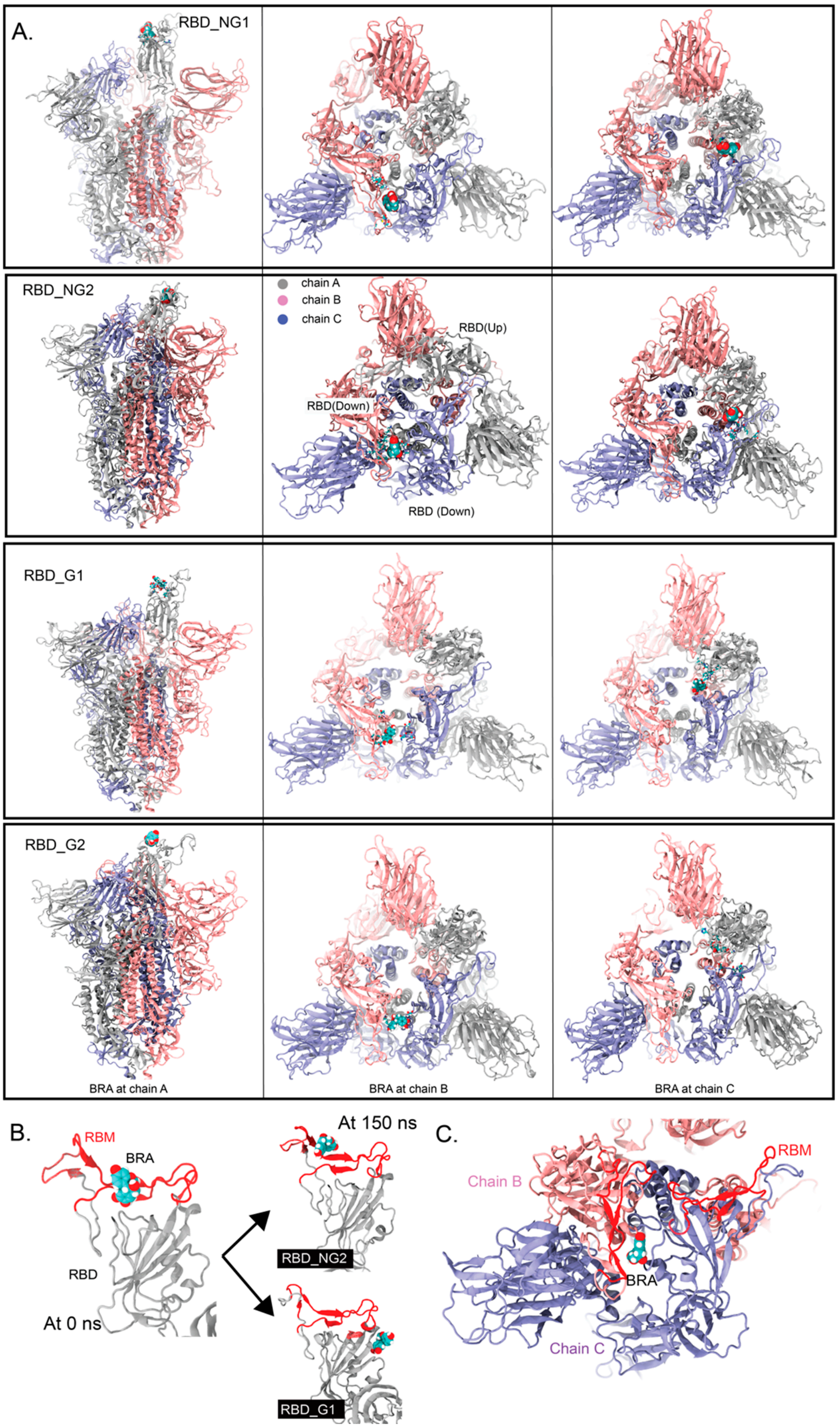

Figure S5. Final snapshots of all RBD_NG and RBD_G systems (150 ns) where BRA is shown in van der Waals format. Chain A is shown in the side view, while chain B and C are in the top view. Chain A, B, and C are coloured in gray, pink, and ice blue, respectively. (B) Reorientation of BRA in RBD_NG2 and RBD_G1. (C) The location of BRA in chain B and C.

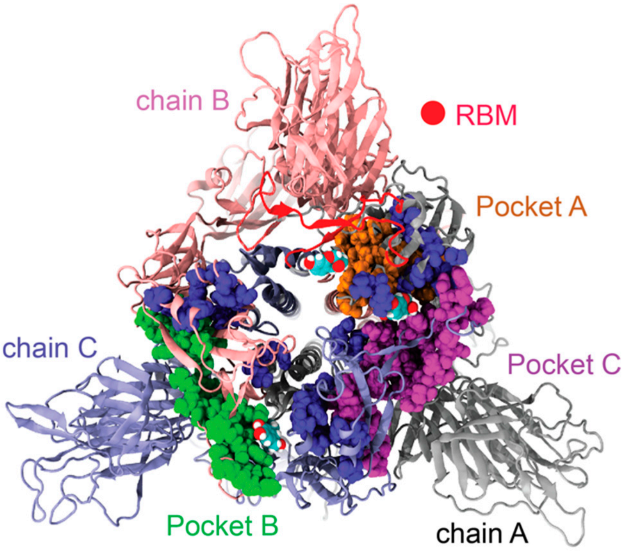

Figure S6. Locations of LA pocket [1] (violet surface) and pocket A-B (orange, magenta, and green surfaces) identified by a recent work [2]. The locations of BRAs in all chains are also displayed. All BRAs are labelled in van der Waals format. RBM of RBD-up chain A is shown in red.

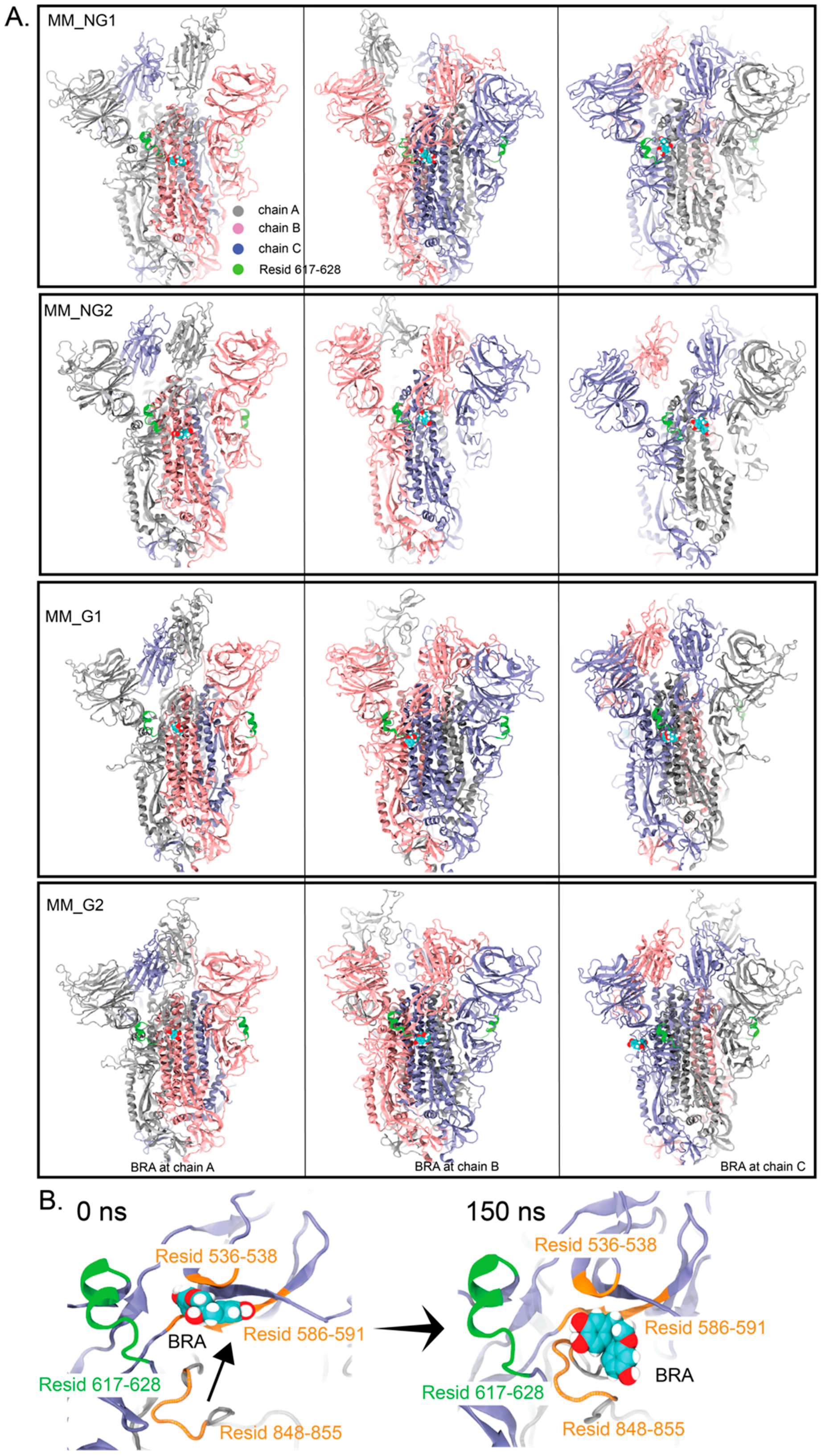

Figure S7. (A) Final snapshots of all MM_NG and MM_G systems (150 ns) where BRA is shown in van der Waals format. Chain A, B, and C are coloured in gray, pink, and ice blue, respectively. (B) Binding location of BRA close to residue 617-628 (green ribbon) where BRA is pinched by orange ribbons (residue 536-538, 586-591, and 848-855).

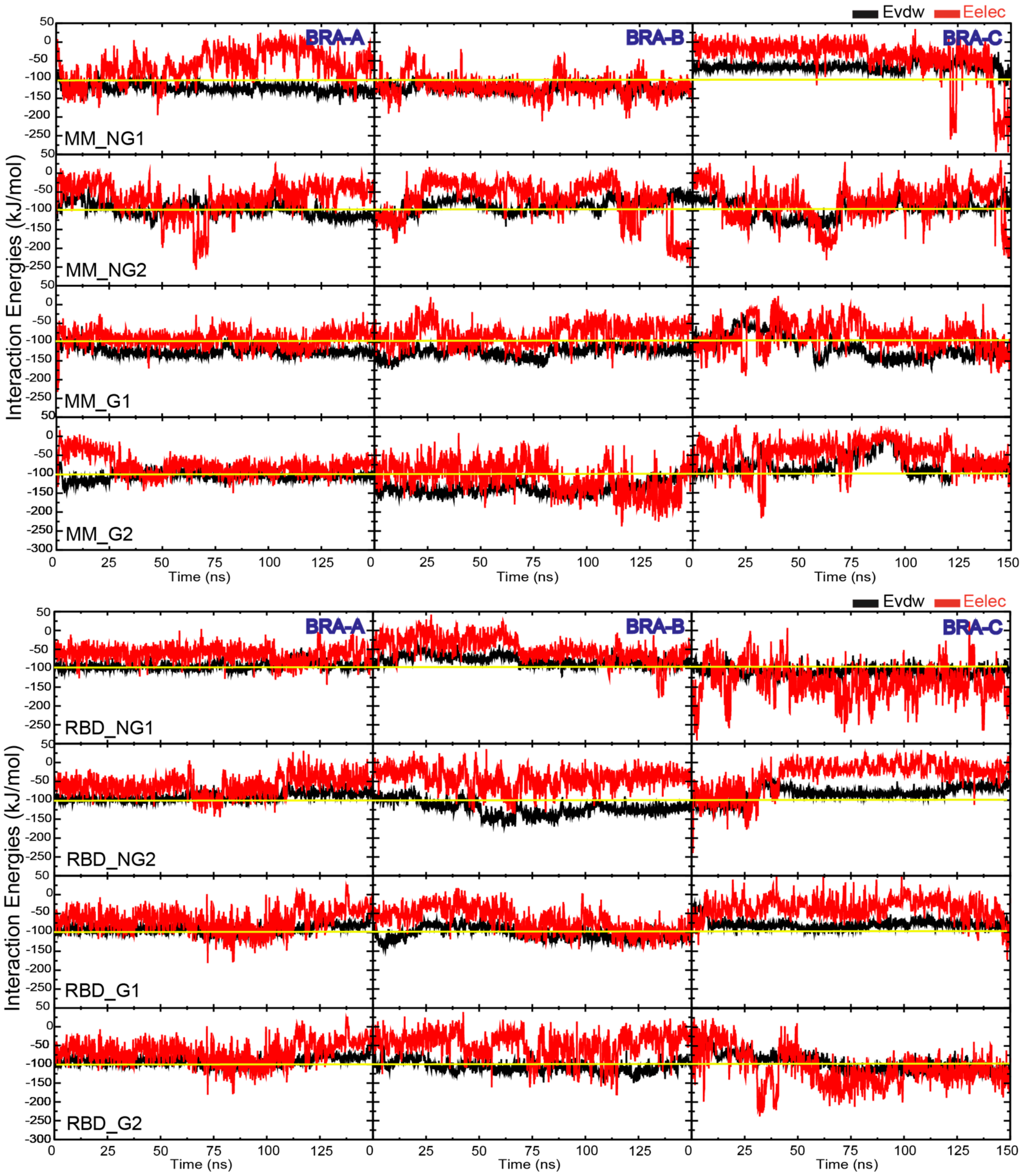

Figure S8. Vdw (black) and Electrostatic (red) interaction energies (kJ/mol) of each system.

Table S1. Gold scores of brazilin (BRA) binding to spike protein at MM and RBD pockets.

Chain	MM pocket	
	G	NG
A	46.31	54.99
B	51.79	47.53
C	39.73	41.37
	RBD pocket	
A	37.69	43.14
B	40.95	37.73
C	35.97	34.70

Table S2. Average number of hydrogen bonds between the Furin cleavage site (residue 682-685 (RRAR)) and glycans.

**System**	**Number of Hydrogen Bonds between RRAR (furin site) with glycans**		
	**BRA-A**	**BRA-B**	**BRA-C**
WT_G1	0.50±0.88	0.06±0.33	0.00±0.00
WT_G2	0.08±0.35	0.07±0.35	0.18±0.58
MM_G1	0.15±0.52	0.00±0.06	0.01±0.07
MM_G2	0.04±0.23	0.14±0.41	0.01±0.09
RBD_G1	0.31±0.67	0.00±0.06	0.00±0.04
RBD_G2	0.02±0.14	0.08±0.31	0.00±0.00

Table S3. Interaction energies (kJ/mol) with standard deviation between BRA and protein. The data after 100 ns were used to calculate the interaction energies using MMPBSA [3].

**System**	**Ligand**								
	**BRA-A**			**BRA-B**			**BRA-C**		
	ΔE_{vdw}	ΔE_{Elec}	**Total Energy**	ΔE_{vdw}	ΔE_{Elec}	**Total Energy**	ΔE_{vdw}	ΔE_{Elec}	**Total Energy**
**MM_NG1**	-127.63±10.47	-45.27±36.53	-172.90±39.82	-125.04±12.34	-120.40±23.82	-245.44±27.39	-63.37±16.30	-82.96±76.10	-146.34±82.93
**MM_NG2**	-106.26±15.75	-45.43±23.30	-151.69±27.49	-76.50±15.39	-96.46±71.50	-172.96±65.61	88.01±12.35	-67.02±46.80	-155.02±58.56
**MM_G1**	-126.74±57.10	-93.08±67.51	-219.82±24.26	-102.84±9.24	-46.78±24.53	-149.62±23.85	-77.40±14.48	-49.65±24.06	-127.05±28.21
**MM_G2**	-102.66±8.90	-83.03±16.68	-185.69±16.95	-122.28±21.21	-132.04±42.29	-254.32±48.29	-83.30±14.01	-59.65±28.96	-142.95±27.36
**RBD_NG1**	-88.79±11.97	-70.29±23.38	-159.07±22.36	-85.61±9.94	-71.26±25.34	-156.87±25.97	-109.34±11.50	-139.47±41.75	-248.81±38.38
**RBD_NG2**	-81.64±10.88	-44.54±23.45	-126.19±28.06	-121.98±9.82	-38.93±19.66	-160.91±20.28	-71.40±12.44	-15.74±18.61	-87.14±19.03
**RBD_G1**	-51.78±14.11	-29.34±21.24	-81.13±27.18	-59.06±9.01	-53.53±23.68	-112.60±22.55	-77.39±8.63	-36.99±33.88	-114.39± 35.11
**RBD_G2**	-83.52±13.12	-50.85±30.17	-134.36±34.42	-90.22±16.42	-19.97± 30.17	-110.19± 30.95	-106.36± 12.53	-116.51±23.88	-222.87±23.80

Table S4. Average number of hydrogen bonds between BRA and glycans with standard deviations.

**System**	**Number of Hydrogen Bonds between BRA with glycans**		
	**BRA-A**	**BRA-B**	**BRA-C**
MM_G1	0.00±0.06	0.53±0.51	0.84±0.91
MM_G2	0.00±0.03	0.08±0.27	0.13±0.37
RBD_G1	0.02±0.14	0.96±1.00	0.84±0.91
RBD_G2	0.00±0.00	1.10±0.88	0.01±0.10

Table S5. Percentages of secondary structure of 617-628 loop in Apo S protein and BRA-MM bound S protein.

System	Secondary structure (%)	
	helix	Coil
Apo S protein	34.33	65.67
BRA-MM bound S protein	38.89	61.11

References

(1) Toelzer, C.; Gupta, K.; Yadav, S. K. N.; Borucu, U.; Davidson, A. D.; Kavanagh Williamson, M.; Shoemark, D. K.; Garzoni, F.; Staufer, O.; Milligan, R.; et al. Free fatty acid binding pocket in the locked structure of SARS-CoV-2 spike protein. *Science* **2020**, *370* (6517), 725-730. DOI: 10.1126/science.abd3255 From NLM Medline.

(2) Cheng, R. L.; Quirante, J. C.; Vargas, L. E. Z.; Gatchalian, A. F.; Nellas, R. B. Complementary Pocket and Network-Based Approach to Search for Spike Protein Allosteric Pocket Sites. *ACS omega* **2023**, *8* (48), 45313-45325.

(3) Miller, B. R., 3rd; McGee, T. D., Jr.; Swails, J. M.; Homeyer, N.; Gohlke, H.; Roitberg, A. E. MMPBSA.py: An Efficient Program for End-State Free Energy Calculations. *J Chem Theory Comput* **2012**, *8* (9), 3314-3321. DOI: 10.1021/ct300418h From NLM PubMed-not-MEDLINE.
